# Supplementary figures and images for: Population history and genome wide association studies of birth weight in a native high altitude Ladakhi population
Source: PLoS One. 2022 Sep 20;17(9):e0269671. doi: 10.1371/journal.pone.0269671 (PMC9488766; doi:10.1371/journal.pone.0269671)

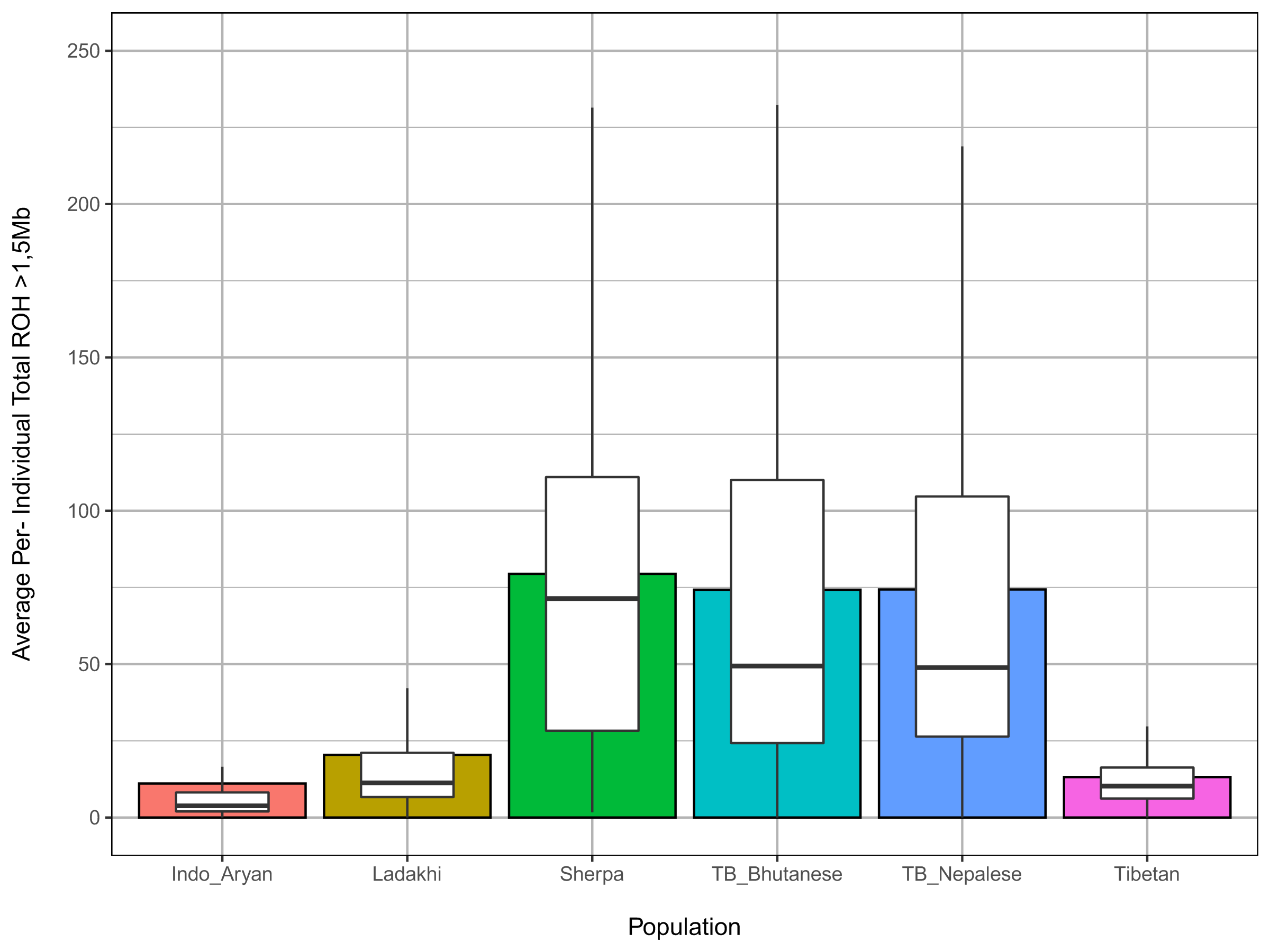

Supplement: S1 Fig — The plot shows the average total length of ROH in each of the populations. The coloured bar shows the arithmetic mean, and the box plots are normal box plots. (TIF) [file pone.0269671.s001.tif]

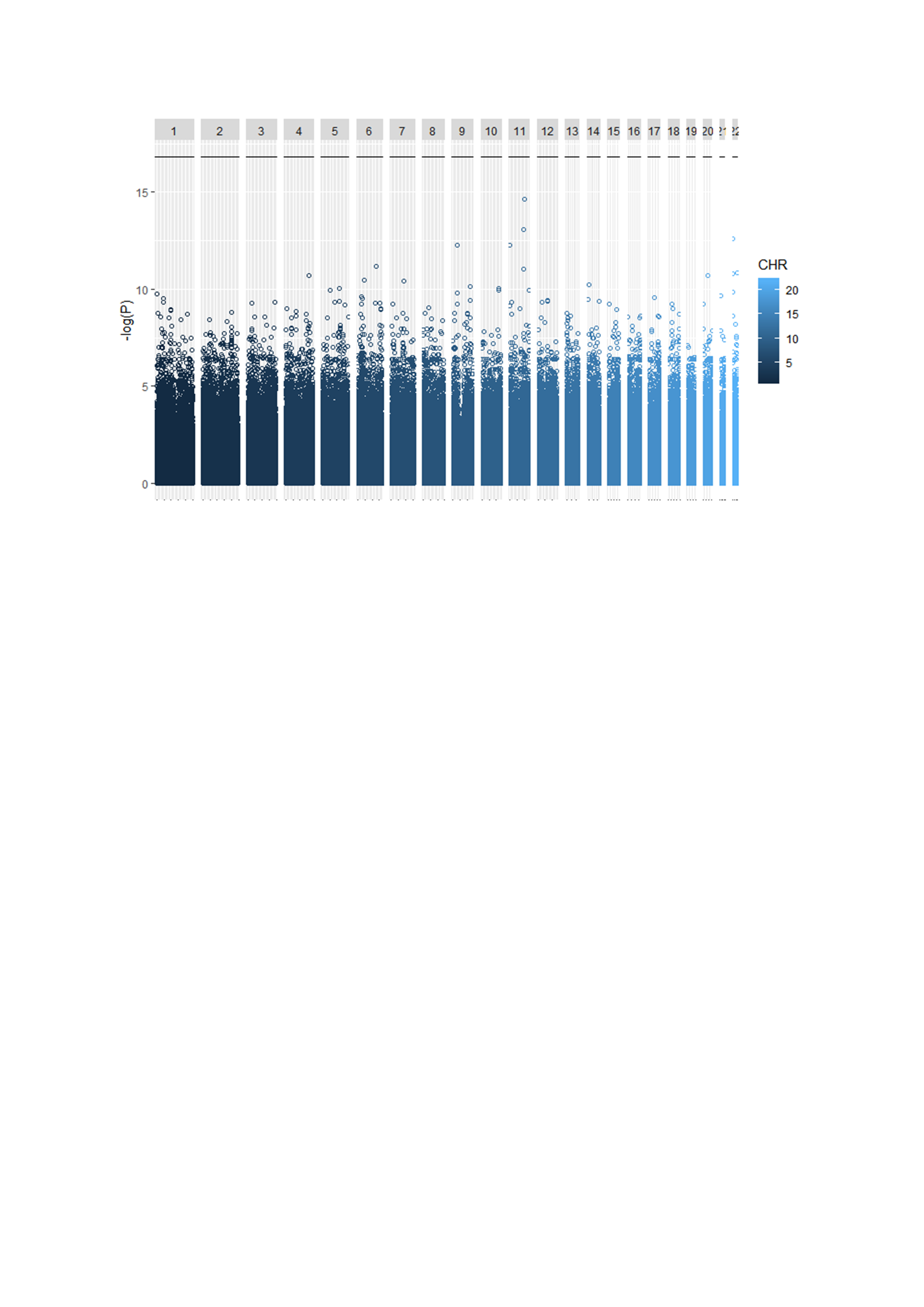

Supplement: S2 Fig — (TIF) [file pone.0269671.s002.tif]
