# Supplementary material for: Population history and genome wide association studies of birth weight in a native high altitude Ladakhi population
Source: PLoS One. 2022 Sep 20;17(9):e0269671. doi: 10.1371/journal.pone.0269671 (PMC9488766; doi:10.1371/journal.pone.0269671)
Supplement: Attachment — Submitted filename: Response to reviewers.docx [file pone.0269671.s003.docx]

*Elizabeth Garrett Anderson Institute for Women’s Health,*

*University College London*

*Wednesday 3^rd^ August 2022*

Dear Editor,

Our thanks to the reviewers for their insightful comments. Please find our responses below in bold and reflected in the amended manuscript.

**Reviewer #1**

**Apologies. Misprints corrected**

The expression 'mechanistic study' is philosophically charged. It would be necessary to explain why you consider genomic studies as 'mechanistic'.

**Wording adjusted to better explain meaning**

The absence of genome wide significance for the variants should deserve a deeper discussion, especially in regard to future studies.

**Agreed and language amended including reference to future studies and more detail inn discussion**

**Reviewer #2**

1) according to recent obstetrical ultrasonography FGR is defined by a Delphi consensus (Gordjin UOG 2016) on the basis of severity of growth restriction (i.e. < 3 centile) or association of a birthweight < 10 and concomitant Doppler changes in fetal hemodynamics. Since I guess that Doppler data analysis are not available in the study population Authors should include a sub analysis of newborn with a birthweight < 3 to show whether their hypothesis fits also in such subjects

**We now reference this definition (line 52) and the number of <3rd centile babies**

2)it is not clear how Authors controlled for other potential confounding variables that influence fetal growth such as alimentation, associated maternal diseases occurring in pregnancy, anemia,

**As part of the phenotype study, we previously published associated features are reported. Almost all Ladakhi women studied were meat eaters, rather than vegetarian. Anaemia was not common (<10% of entire cohort) and women had very few co-morbidites (this information has been added to Table 1). Further data from our previous paper is added to Table 1 (line 108)**.

**We could not reliably measure all in the field and some (eg [Hb]) varies across pregnancy. Such issues will not confound even if directly and mechanistically related to the gene variants under study, they will contribute to the observed genetic association but we have included in our discussion a line stating that introducing some environmental variation, may weaken the power of the study to identify genetic markers.**

3)I suggest adding a table sub grouping women according to the ethnicity (Tibetan and Ladakhi) and performing an univariate analysis of maternal and obstetrical characteristics

**We had very few Tibetan women recruited in the overall study (n=10) (reported in Table 1) and those included in the birth weight analysis were of Ladakhi genomic ancestry by GWAS.**

4)the limitations of the study should be reported

**Thank you**. **We have made this more apparent within the body of text.**

**Reviewer #**3

1. Since birth weight is an important parameter of the study, a better specification of Fetal Growth Restriction with ultrasound during pregnancy according to the Delphi consensus and its association with the birthweight could be included, if available.

**Please see comments above for reviewer 2 and we agree and have included in discussion**

2. Since fetal development is strictly linked to the maternal environment (nutrition, physical activity, individual and community stress) and not only to the altitude considered in the study and since mothers completed a comprehensive questionnaire of their pregnancy history, but it would also be interesting to make one or more tables showing the data. It should include also the relevant medical history when present.

**Please see comments above for reviewer 2 and we agree and have included in discussion**

3. It would be interesting to compare different ethnicities and maternal medical and obstetrical histories.  **Our study concentrated on Ladakhi pregnant women to better ascertain in this population genetic variants. The Number of women of different ethnicities was small in this study but would be interesting to review. Equally, the number of pregnant women recruited with significant medical/ obstetric complications was limited but would beef interest in the future.**

4. The limitations of the study should be better presented. **Thank you. Discussion adjusted.**

Yours sincerely (on behalf of all authors),

Sara Hillman

Associate Professor and Maternal Fetal Consultant University College London

[Sara.hillman@ucl.ac.uk](mailto:Sara.hillman@ucl.ac.uk)
